# Dissecting C−H∙∙∙π and N−H∙∙∙π Interactions in Two Proteins Using a Combined Experimental and Computational Approach

**DOI:** 10.1038/s41598-019-56607-4

**Published:** 2019-12-27

**Authors:** Jia Wang, Lishan Yao

**Affiliations:** 1 Key Laboratory of Biofuels, Qingdao Institute of Bioenergy and Bioprocess Technology, Chinese Academy of Sciences, Qingdao, 266101 China; 2 Shandong Provincial Key Laboratory of Synthetic Biology, Qingdao Institute of Bioenergy and Bioprocess Technology, Chinese Academy of Sciences, Qingdao, 266101 China; 3 University of Chinese Academy of Sciences, Beijing, 100049 China

**Keywords:** Biochemistry, Biophysics

## Abstract

C−H∙∙∙π and N−H∙∙∙π interactions can have an important contribution for protein stability. However, direct measurements of these interactions in proteins are rarely reported. In this work, we combined the mutant cycle experiments and molecular dynamics (MD) simulations to characterize C−H∙∙∙π and N−H∙∙∙π interactions and their cooperativity in two model proteins. It is shown that the average C−H∙∙∙π interaction per residue pair is ~ −0.5 kcal/mol while the N−H∙∙∙π interaction is slightly stronger. The triple mutant box measurement indicates that N−H∙∙∙π∙∙∙C−H∙∙∙π and C−H∙∙∙π∙∙∙C−H∙∙∙π can have a positive or negative cooperativity. MD simulations suggest that the cooperativity, depending on the local environment of the interactions, mainly arises from the geometric rearrangement when the nearby interaction is perturbed.

## Introduction

X−H∙∙∙π interactions in biomolecules, where X can be C, N, O, or S are weak and attractive interactions between the X−H component and aromatic groups. The high incidence in biomolecules makes X−H∙∙∙π interactions an important contributor to the structure and function, and has led to an increasing number of theoretical and experimental studies devoted to characterization of such interactions^1–10^. Theoretical studies show that N−H∙∙∙π, O−H∙∙∙π and C−H∙∙∙π can have very different optimum geometries, with the interaction strength order O−H∙∙∙π > N−H∙∙∙π > C−H∙∙∙π^4,11^. The S−H∙∙∙π interaction can be weaker^9^ or stronger^12^ than O−H∙∙∙π, but is generally stronger than N−H∙∙∙π and C−H∙∙∙π^9,12^. The computational interaction energy of the indole-benzene dimer where the N−H∙∙∙π interaction exists can reach −5.2 kcal/mol^13^. The computational interaction energies between benzene and CH_4_, NH_3_, H_2_S, and H_2_O are −1.4, −2.5, −2.9 and −3.0 kcal/mol, respectively^9^. The computational binding energies between indole and CH_4_, NH_3_, H_2_S, and H_2_O are −2.0, −2.6, −4.9 and −3.6 kcal/mol, respectively^12^. The importance of the S−H∙∙∙π and C−H∙∙∙π interactions in proteins has also been highlighted by their occurrence in the PDB database search^8,14,15^. The C−H∙∙∙π interaction has been observed directly in proteins by nuclear magnetic resonance (NMR) spectroscopy methods where the across C−H∙∙∙π J-coupling is detected^16^. Quantification of C−H∙∙∙π in calix[4]pyrrole receptors yields a magnitude of −1 kcal/mol^17^. The C−H∙∙∙π interaction in benzene−methane, ethane, propane, and butane, increases monotonically from −1.1 to −2.7 kcal/mol^18–20^. The measurement of C−H∙∙∙π interactions in a cyclohexylalanine−phenylalanine pair in the core of a synthetic peptide indicates that each C−H∙∙∙π contact can contribute about −0.7 kcal/mol to peptide stability^21^. In real proteins, C−H∙∙∙π mainly occurs between an aliphatic side chain and an aromatic ring, or between two aromatic rings^14^. Although C−H∙∙∙π interactions are well documented in proteins^1^, direct measurements of C−H∙∙∙π and N−H∙∙∙π strength in proteins are scarce.

Another important issue about X−H∙∙∙π interactions is their cooperativity. Cooperativity is a central concept for understanding molecular recognition and supramolecular self-assembly^22^. By forming networks of weak interactions that compete against the entropy of flexible polypeptides, proteins fold into their biologically functional three-dimensional structures^23^. As a part of the interaction network, how X−H∙∙∙π interactions coexist and cooperate in proteins is an important question. Only a few studies have addressed the X−H∙∙∙π cooperativity, mainly in small molecules. The cooperativity of C−H∙∙∙π interactions in small molecules is studied using molecular torsional balances^24^. The average C−H∙∙∙π interaction strength increases as more C−H∙∙∙π pairs are formed, suggesting a positive cooperativity. This is opposite to the findings of an earlier computational study where the negative cooperativity is concluded for the same complexes^25^. The C−H∙∙∙π and N−H∙∙∙π cooperativity in proteins remains largely unexplored.

In this work, we attempt to measure the C−H∙∙∙π and N−H∙∙∙π interactions in protein GB3 and staphylococcal nuclease (SNase). GB3 is the third immunoglobulin binding domain of protein G, a model protein that has been extensively studied^26^. SNase is an enzyme that hydrolyzes nucleotides in DNA or RNA. A stable mutant of SNase, Δ + PHS, is selected as the test system^27^. It is found experimentally that the C−H∙∙∙π interaction on average is about −0.5 kcal/mol and the N−H∙∙∙π interaction on average is about −0.6 kcal/mol. N−H∙∙∙π…C−H∙∙∙π and C−H∙∙∙π…C−H∙∙∙π can have different cooperativities. Molecular dynamics (MD) simulations can reproduce N−H∙∙∙π and C−H∙∙∙π interactions and their cooperativities with reasonable accuracy. Geometric parameters that are important for C−H∙∙∙π and N−H∙∙∙π interactions are discussed. Their contribution to cooperativity is illustrated. With the combination of experimental and computational results, a better view of C−H∙∙∙π, N−H∙∙∙π and their cooperativity is obtained.

## Results

### Experimental C−H∙∙∙π and N−H∙∙∙π interaction energies

**Figure 1:**
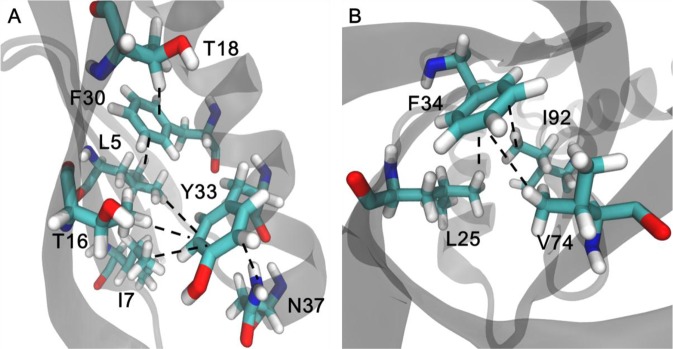
Putative C−H∙∙∙π and N−H∙∙∙π interactions in GB3 (panel A, pdb code: 2OED) Δ + PHS (pdb code: 3BDC). L5 and T18 interact with F30 whereas L5, I7, T16, and N37 interact with Y33 in GB3. L25, V74 and I92 are in contact with F34 in Δ + PHS.

Based on the X-ray crystal structures, a series of X−H∙∙∙π interactions can be identified in GB3 and Δ + PHS (pdb code: 2OED and 3BDC, respectively). GB3 has five residue pairs that may form C−H∙∙∙π interactions, L5−F30, T18−F30, L5−Y33, I7−Y33, and T16−Y33, and one residue pair N37−Y33 that can form the N−H∙∙∙π interaction (Fig. 1A). Δ + PHS has three C−H∙∙∙π interaction residue pairs, L25−F34, V74−F34, I92−F34 (Fig. 1B). All these C−H∙∙∙π interactions are between a methyl group and an aromatic ring. A total of nine C−H∙∙∙π interactions were characterized, including L5−F30, T18−F30, L5−Y33, I7−Y33, T16−Y33, and T16−F33 of GB3, and L25−F34, V74−F34, and I92−F34 of Δ + PHS. Two N−H∙∙∙π interactions N37−Y33 and N37−F33 in GB3 were also measured. Furthermore, the introduction of triple mutant boxes (TMBs) generates additional 16 C−H∙∙∙π and 4 N−H∙∙∙π pairs (Table 1). Therefore, a total of 25 C−H∙∙∙π and 6 N−H∙∙∙π interactions were measured.

**Table 1:** Experimental interaction energies of C−H∙∙∙π and N−H∙∙∙π interactions from double mutant cycle analysis.

Protein	Residue pair	Interaction (kcal/mol)	Protein	Residue pair	Interaction (kcal/mol)
**C−H∙∙∙π**					
GB3 (WT)	L5−F30	−0.81 ± 0.05	GB3 (N37A)	I7−Y33	0.11 ± 0.06
GB3 (WT)	L5−Y33	−0.65 ± 0.06	GB3 (N37A)	T16−Y33	−0.85 ± 0.06
GB3 (WT)	I7−Y33	−0.13 ± 0.06	GB3 (Y33F-N37A)	T16−F33	−0.29 ± 0.06
GB3 (WT)	T16−Y33	−0.70 ± 0.05	Δ + PHS (WT)	L25−F34	−0.41 ± 0.05
GB3 (WT)	T16−F33	−0.68 ± 0.05	Δ + PHS (WT)	V74−F34	−0.53 ± 0.07
GB3 (WT)	T18−F30	−0.08 ± 0.05	Δ + PHS (WT)	I92−F34	−0.46 ± 0.06
GB3 (L5V)	T16−Y33	−0.29 ± 0.06	Δ + PHS (L25V)	V74−F34	−0.83 ± 0.05
GB3 (L5V)	T18−F30	0.09 ± 0.06	Δ + PHS (L25V)	I92−F34	−0.84 ± 0.06
GB3 (I7V)	T16−Y33	−0.25 ± 0.06	Δ + PHS (V74A)	L25−F34	−0.72 ± 0.04
GB3 (T16A)	L5−Y33	−0.26 ± 0.06	Δ + PHS (V74A)	I92−F34	−0.51 ± 0.05
GB3 (T16A)	I7−Y33	0.31 ± 0.05	Δ + PHS (I92V)	L25−F34	−0.81 ± 0.08
GB3 (T18A)	L5−F30	−0.65 ± 0.06	Δ + PHS (I92V)	V74−F34	−0.59 ± 0.07
GB3 (N37A)	L5−Y33	−0.34 ± 0.06			
**N−H∙∙∙π**					
GB3 (WT)	N37−Y33	−0.68 ± 0.05	GB3 (T16A)	N37−Y33	−0.86 ± 0.06
GB3 (L5V)	N37−Y33	−0.37 ± 0.07	GB3 (Y33F)	N37−F33	−0.83 ± 0.06
GB3 (I7V)	N37−Y33	−0.45 ± 0.06	GB3 (Y33F-T16A)	N37−F33	−0.15 ± 0.06

The folding free energies Δ*G* of all proteins were derived from the denaturation curves. The values of [*D*]_50%_, *m* values for the wild type and mutant proteins are given in Supplementary Table S1. The magnitude of noncovalent interactions in the two proteins GB3 and Δ + PHS was obtained using the double mutant cycle (DMC) analysis^28^. The values of C−H∙∙∙π interactions are shown in Table 1, ranging from +0.31 (unfavorable) to −0.85 (favorable) kcal/mol, with 22 out of 25 showing favorable interactions. The three small positive interaction energies may come from the secondary interactions, i.e., the interaction changes from the surrounding residues caused by mutations (a caveat of the DMC experiment). The residual secondary interactions can contribute to the measured XH∙∙∙π energy which may change the sign of the energy (to repulsive) if it is small. The interaction energy of N−H∙∙∙π ranges from −0.15 to −0.86 kcal/mol.

According to DMC, it is preferable to mutate the two side chains *x* and *y* in the X−H∙∙∙π pair to alanine residues to completely remove the interactions between the two. However, eliminating an aromatic residue in a protein core can be detrimental to protein stability. Instead, we only mutated the aromatic side chain (*y*) to a leucine (*y*′) which is still hydrophobic but disrupts the X−H∙∙∙π interaction (see more details in Materials and Methods). For the X−H component (*x*), conservative mutations are introduced (*x*′) to remove the X−H∙∙∙π interaction and maintain the protein folding at the same time. These mutations may create residual pairwise side chain interactions in *x*′*y*′, *xy*′, and *x*′*y*. Furthermore, for a residue like leucine (for example, in L5−F30) which has two CH3 and one CH, it can form multiple C−H∙∙∙π interactions which complicate the interpretation of the experimental results. These problems can be solved with the assist of MD simulations.

### Benchmark of MD simulations

**Figure 2:**
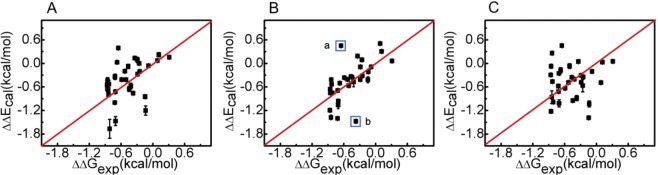
Correlation between the experimental and calculated interaction energies from different force fields. (**A**) Amberff99SB, (**B**) Charmm27, and (**C**) Gromos53a6. The RMSDs from the experimental values are 0.41, 0.27 (excluding two outliers, a: L5−F30 in GB3(T18A), b: N37−Y33 in GB3(L5V)), and 0.47 kcal/mol, respectively. The red line is *y* = *x*.

MD simulations were performed for all the experimentally measured mutants with three commonly used force fields, Amber99sb^29^, Charmm27^30^, and Gromos53a6^31^. The experimental C−H∙∙∙π and N−H∙∙∙π interaction energies were used as a benchmark to evaluate the accuracy of different force fields. The root mean square deviation (RMSD) between the experimental and predicted X−H∙∙∙π interactions was calculated:

1 $$RMSD=\sqrt{\frac{\mathop{\sum }\limits_{i=1}^{N}{(\Delta \Delta {G}_{\exp }-\Delta \Delta {E}_{MD})}^{2}}{N}}$$

where *N* is 31, the total number of measured residue pairs that form X−H∙∙∙π interactions, ΔΔ*G*_exp_ is the experimental X−H∙∙∙π interaction energy, and ΔΔ*E*_*MD*_ is the calculated interaction energy. Charmm27 appears to perform better than the other two force fields. Its RMSD value is 0.27 kcal/mol (after removing two apparent outliers), while the RMSDs of Amber99sb and Gromos53a6 are 0.41 and 0.47 kcal/mol, respectively (Fig. 2). Thus, the trajectories produced using Charmm27 were selected for further analyses.

### Geometric parameters of C−H∙∙∙π and N−H∙∙∙π interactions

**Figure 3:**
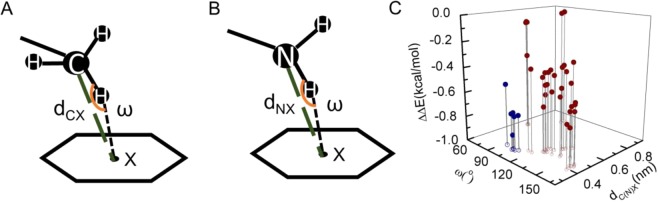
Geometric parameters and computational interaction energies for C−H∙∙∙π and N−H∙∙∙π interactions. (**A**) Schematic diagram of C−H∙∙∙π. The center-of-mass of the π-system is indicated by the point X. *d*_CX_ is distance between the center-of-mass of the methyl group and that of the aromatic ring, ω is the angle ∠C−H−X. (**B**) Schematic diagram of N−H∙∙∙π. (**C**) Computational ΔΔ*E* energy scatter plot with *d*_CX_ or *d*_NX_ and *ω*. ΔΔ*E* is the interaction energy between the methyl group and the aromatic group (red) and those between the amide group and the aromatic group (blue).

The reasonable correlation between the interaction energies from MD simulations and experiments encourages us to investigate the geometric parameters that are important for C−H∙∙∙π and N−H∙∙∙π interactions. The pairwise interaction energy ΔΔ*E*_CH3∙∙∙π_ between a CH3 group and a aromatic ring was calculated for all the C−H∙∙∙π interactions identified above. Two geometric parameters^15^ *d*_CX_ and *ω* are defined for the C−H∙∙∙π interaction, where *d*_CX_ is the distance of the methyl carbon to the center of mass of the aromatic ring (X), and *ω* is the ∠C−H−X angle (Fig. 3A). Since there are three methyl hydrogens, the one with the largest ∠C−H−X angle is defined as *ω*. The same geometric parameters can also be defined for N−H∙∙∙π interactions (Fig. 3B). The 3D plot of (*d*_CX_, *ω*) versus ΔΔ*E*_CH3∙∙∙π_ shows that the geometries with shorter *d*_CX_ and larger *ω* have more negative interaction energies (Fig. 3C). The distance appears to be the most important parameter, with the energy dropping quickly as the distance decreases. Meanwhile, the angle *ω* can also be important. The average Δ*E*_CH3∙∙∙π_ for all the C−H∙∙∙π interactions is −0.36 kcal/mol. The number of N−H∙∙∙π interactions is less than that of C−H∙∙∙π, and they appear to be stronger than C−H∙∙∙π interactions with the same geometric parameters.

### ∆ΔΔ*G*_coop_ from TMB measurements

**Figure 4:**
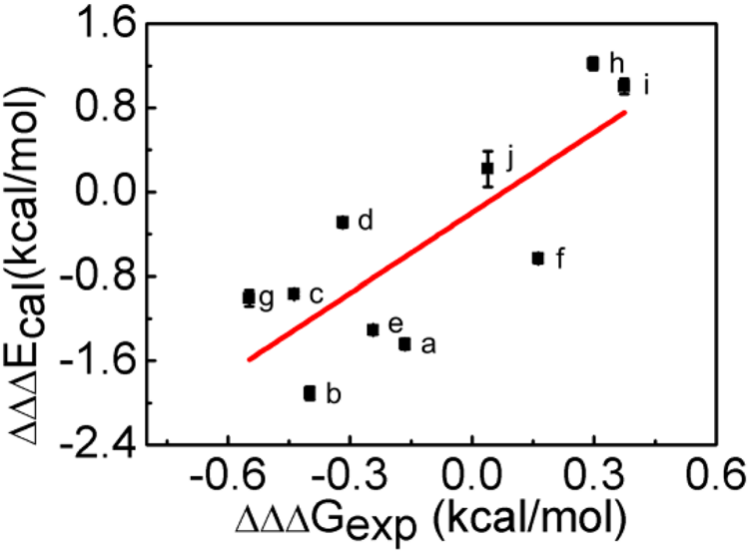
Correlation between the experimental and calculated cooperativity energies (**a**). L5−F30−T18, (**b**) L5−Y33−T16, (**c**) I7−Y33−T16, (**d**) L5−Y33−N37, (**e**) I7−Y33−N37, (**f**) T16−Y33−N37, (**g**) T16−F33−N37, (**h**): L25−F34−V74, (**i**): L25−F34−I92, (**j**): V74−F34−I92). The best fitted line is *y* = 2.54*x* − 0.2, with a correlation coefficient *R* of 0.79. Groups a−g are from GB3 whereas groups h−j are from Δ + PHS.

On the basis of double mutant cycles we had, we established several TMBs to elucidate the cooperativity in C−H∙∙∙π∙∙∙C−H∙∙∙π and C−H∙∙∙π∙∙∙N−H∙∙∙π interactions. In protein GB3, the cooperativity is positive in L5−F30−T18, L5−Y33−T16, I7−Y33−T16, L5−Y33−N37, I7−Y33−N37, and T16−F33−N37, with ∆ΔΔ*G*_coop_ varied from −0.16 to −0.55 kcal/mol (Supplementary Table S2, Fig. 4), suggesting that they are cooperative with each other. In contrast, the C−H∙∙∙π∙∙∙N−H∙∙∙π in T16−Y33−N37 of GB3, and the C−H∙∙∙π∙∙∙C−H∙∙∙π in L25−F34−V74, L25−F34−I92, and V74−F34−I92 of Δ + PHS are anticooperative, with ∆ΔΔ*G*_coop_ varied from 0.04 to 0.37 kcal/mol (Supplementary Table S2). The cooperativity difference in different C−H∙∙∙π∙∙∙C−H∙∙∙π and C−H∙∙∙π∙∙∙N−H∙∙∙π suggests that it depends on the local interaction network.

### Cooperativity mechanism from MD simulations

**Figure 5:**
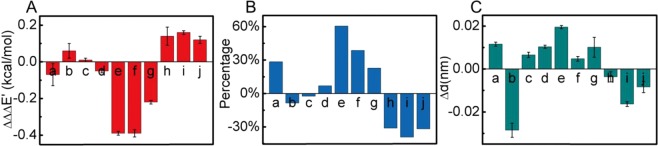
(**A**) Calculated cooperativity energy ∆ΔΔ*E*′ for ten side chain groups (same as those in Fig. 4). (**B**) Percentagewise ∆ΔΔ*E*′ defined as ∆ΔΔ*E*′, divided by the average of the two C−H∙∙∙π/C−H∙∙∙π or C−H∙∙∙π/N−H∙∙∙π interactions. (**C**) *∆d*, the first C−H∙∙∙π or N−H∙∙∙π distance change when the second C−H∙∙∙π or N−H∙∙∙π is removed.

The ∆ΔΔ*G*_coop_ are in a good correlation with the computational ∆ΔΔ*E* (cooperativity energy, see more details in Materials and Methods), although the absolute value of ∆ΔΔ*E* is generally larger than that of ∆ΔΔ*G*_coop_ (Fig. 4). One likely cause is that the entropic contribution, which is not calculated in MD simulations, may offset the large change of ∆ΔΔ*E*. The entropy calculation is far more difficult (less reliable) and thus not pursued. As discussed above, the residual interactions caused by the experimental non-alanine mutations complicate the interpretation of ∆ΔΔ*G*_coop_. To solve this problem, we rebuilt TMBs by mutating the three side chains, for example L25, F34, and V74 in L25−F34−V74, to alanines systematically in MD simulations. The cooperativity energy ∆ΔΔ*E*′ was calculated for the residue groups listed above with the same procedure (Fig. 5A). The cooperativity from ∆ΔΔ*E*′ generally agrees with that from ∆ΔΔ*E*, except that L5−Y33−T16 and I7−Y33−T16 show a weak negative instead of positive cooperativity.

The cooperativity energy ∆ΔΔ*E*′ varies from −0.39 to 0.16 kcal/mol (Fig. 5A). Although they appear to be small, the percentagewise ∆ΔΔ*E*′ (∆ΔΔ*E*′ divided by the average of the two C−H∙∙∙π interactions in C−H∙∙∙π∙∙∙C−H∙∙∙π or the average of the C−H∙∙∙π and the N−H∙∙∙π interaction energy in C−H∙∙∙π∙∙∙N−H∙∙∙π) can vary from −40% (cooperative) to +60% (anticooperative) (Fig. 5B). So it is obvious that cooperativity can be very important for C−H∙∙∙π and N−H∙∙∙π interactions in an interaction network. To further understand the origin of cooperativity, the geometric changes in the TMB are investigated. It is known that *d*_CX_ or *d*_NX_ (Fig. 3) is an important parameter for C−H∙∙∙π or N−H∙∙∙π. Using L5−Y33−T16 as an example, Δ*d*, the change of *d*_CX_, was calculated by

2 $$\Delta d={d}_{CX\_WT}-{d}_{CX\_MUT}$$

where *d*_CX_WT_ is *d*_CX_ between the methyl of L5 and the aromatic side chain of Y33 in the wild type, and *d*_CX_MUT_ is *d*_CX_ in the single mutant T16A. A similar Δ*d* can be defined for C−H∙∙∙π∙∙∙N−H∙∙∙π interactions. Δ*d* was calculated for 10 residue groups shown in Fig. 5. The positive Δ*d* corresponds to the increase of the first C−H∙∙∙π (or N−H∙∙∙π) distance when the aliphatic side chain of the second C−H∙∙∙π (or N−H∙∙∙π) is mutated to alanine. In other words, removing the second C−H∙∙∙π (or N−H∙∙∙π) interaction weakens the first C−H∙∙∙π (or N−H∙∙∙π) interaction, suggesting a positive cooperativity. For 9 out of 10 groups, the distance change Δ*d* predicts the cooperativity consistent with the interaction energy result (Fig. 5B,C), indicating that the cooperativity in C−H∙∙∙π∙∙∙C−H∙∙∙π or C−H∙∙∙π∙∙∙N−H∙∙∙π mainly arises from the geometric rearrangement.

## Discussion

DMC experiments are commonly used to measure residue−residue interactions, such as salt bridges and hydrogen bonds^32,33^. However, measuring C−H∙∙∙π interactions in the protein interior using DMC can be challenging because removing an aromatic side chain can destabilize and even unfold the protein. In this work, we only mutate the aromatic residue to leucine which maintains the protein folding and removes the C−H∙∙∙π interaction. Two very stable proteins GB3 and Δ + PHS were selected for the purpose. One caveat of the F or Y to L mutation is that residual interactions with leucine complicate the data interpretation. Molecular dynamics simulations were used to decompose the various contributions and help us focus on the C−H∙∙∙π interactions. The good agreement between experimental and computational interaction energies validates the procedure which provides important insights about the C−H∙∙∙π and N−H∙∙∙π interactions.

The energy of C−H∙∙∙π interactions obtained from the DMC experiments of two proteins in this work is smaller than ~ −0.9 kcal/mol, with an average of ~ −0.5 kcal/mol. This C−H∙∙∙π interaction strength is generally weaker than those reported for small molecules^17–21^. It is likely that different interactions compete with each other in proteins so that the C−H∙∙∙π interaction of a specific residue pair is not in an optimum geometry. This is evident from the interaction energy landscape of methyl−aromatic ring pair (Fig. 3). The lower corner, with *d*_CX_ of ~0.4 nm and *ω* of ~165°, has the lowest interaction energy in the plot. But many C−H∙∙∙π pairs are clustered around *d*_CX_ of ~0.4−0.6 nm and *ω* of ~120°−150°. The optimal *d*_CX_ of 0.4 nm is close to the distance obtained from the quantum mechanical calculations^9^. For C−H∙∙∙π pairs with larger *d*_CX_, the C−H group moves away from the top of the aromatic ring to form a side-by-side configuration which has an optimal *d*_CX_ of ~0.5 nm, as suggested from the QM calculations^9^. The non-optimum geometry also implies that different C−H∙∙∙π interactions with the same aromatic ring are interdependent. A small perturbation of one C−H∙∙∙π pair may affect the geometry of another C−H∙∙∙π nearby which creates the cooperativity effect.

The cooperativity analysis from TMB clearly suggests that the C−H∙∙∙π…C−H∙∙∙π and C−H∙∙∙π…N−H∙∙∙π can be either cooperative or anticooperative (Fig. 4). Although in the experimental TMB analysis, the cooperativity information is contaminated by the residual interactions in the mutants, the computational TMB analysis where the residual interactions are removed suggests that the side chain C−H∙∙∙π and N−H∙∙∙π interactions have a major contribution to the experimentally determined ∆ΔΔ*G* (Figs. 4 and 5). Moreover, the *d*_CX_ or *d*_NX_ distance change Δ*d* is an important indicator for the cooperativity. But when comparing the computational cooperativity energy ∆ΔΔ*E*′ and Δ*d*, the linear correlation between the two is only moderate, suggesting that the distance change is not the only contributor to the cooperativity change. The change of angles such as *ω* may also play a role.

Two simpler cooperativity models were built using two methane and one benzene molecules, with methanes on the same side (MMB) or opposite side (MBM) of the benzene. The cooperativity energies of MMB and MBM models were calculated at the MP2/aug-cc-pvtz level^34^. According to the quantum mechanical (QM) calculations, the cooperativity energy of MMB is 0.74 kcal/mol, indicating that C−H∙∙∙π…C−H∙∙∙π is anticooperative in this model, while the cooperativity energy of MBM is 0.03 kcal/mol, suggesting that there is no cooperativity in this model. Similar to the result in the MD simulations, the geometric reorganization occurs in the MMB model where the two methanes compete for the binding site. No such competition exists in the MBM model where the cooperativity energy is close to zero. The QM calculations highlight the importance of geometric reorganization to cooperativity.

## Conclusion

In this study, we measured the strength of C−H∙∙∙π and N−H∙∙∙π interactions in GB3 and SNase. The C−H∙∙∙π interaction is about 0.3 to −0.9 kcal/mol whereas the N−H∙∙∙π interaction is about −0.2 to −0.9 kcal/mol. The energy decomposition from MD simulations helps determine the C−H∙∙∙π and N−H∙∙∙π interactions for individual methyl−aromatic and amino−aromatic pairs and identify important geometric parameters *d*_C(N)X_ and *ω*. The experimental TMB analysis suggests that the cooperativity of X−H∙∙∙π interactions can be either positive or negative, depending on the local environment. The cooperativity trend is successfully captured by MD simulations where the cooperativity energy can reach ~ −40% to 60% of C−H∙∙∙π or N−H∙∙∙π interactions, highlighting its importance in proteins. The geometric rearrangement is the main cause for the cooperative interactions. It is worth noting that the C−H∙∙∙π and N−H∙∙∙π interactions and the cooperativity were only measured for two proteins GB3 and Δ + PHS. More measurements will be needed to see whether the conclusions also hold for other proteins. But we expect that the mechanism behind the interactions is universal for all protein molecules.

## Materials and Methods

### Protein expression and purification

The wild type and mutants of GB3 and Δ + PHS were prepared with the PCR-based site-directed mutagenesis on vector pET-11b. These plasmids were transformed into the *E*. *coli* strain BL21 (DE3) cells for protein expression. The purification procedure for GB3 and its variants has been described previously^35^. Δ + PHS and its variants were purified using the same procedure as described by Shortle and Meeker^36^.

### Thermodynamic stability measurements

All the denaturation measurements were performed using a HITACHI f-4600 fluorescence Spectrophotometer. Mixtures consisted of up to 6.0 M GdnHCl and 50 µM proteins (final concentration) were incubated for 30 min at 30 °C. The signal intensity at 340 nm for GB3 and 348 nm for SNase was extracted and fitted using the following equation,

3 $$S=\frac{({\alpha }_{N}+{\beta }_{N}[D])+[({\alpha }_{U}+\beta [D])\exp [[m([D]-{[D]}_{50 \% })]]/RT}{1+\exp [m[([D]-{[D]}_{50 \% }/RT}$$

where *S* is the measured Fluo_340nm_ or Fluo_348nm_, *α*_N_ and *α*_U_ are the intercepts and *β*_N_ and *β*_U_ are the slopes of the Fluo_340nm_ or Fluo_348nm_ baselines at low (*N*) and high (*U*) denaturant concentrations, *R* is the Boltzmann constant, *T* is the temperature, [*D*] is the denaturant concentration, [*D*]_50%_ is the denaturant concentration at which the protein is 50% denatured.

### Double mutant cycle analysis

Double mutant cycle (DMC), proposed by Fersht and co-workers, can eliminate the contribution of the secondary interactions and obtain accurate binding energy for the interaction between two residues^37,38^. Double mutant cycles were performed to quantify C−H∙∙∙π interactions and N−H∙∙∙π interactions in this work. To build the DMC, dozens of single and double mutants were prepared. Single mutants included L5V, I7V, T16A, T18A, N37A, F30L, Y33L, Y33F in GB3 and L25V, V74A, I92V, F34L in Δ + PHS. Double mutants contained two substitutions, L5V−F30L, L5V−Y33L, I7V−Y33L, T16A−Y33F, T16A−Y33L, T18A−F30L, N37A−Y33L and N37A−Y33F in GB3, and L25V−F34L, V74A−F34L, I92V−F34L in Δ + PHS. The folding free energy for each mutant was determined from the denaturation curve monitored by fluorescence. The C−H∙∙∙π or N−H∙∙∙π interaction energy with the aromatic ring was then calculated using:

4 $${\Delta \Delta {\rm{G}}}_{xy}=\Delta {G}_{xy}-\Delta {G}_{x^{\prime} y}-\Delta {G}_{xy^{\prime} }+\Delta {G}_{x^{\prime} y^{\prime} }$$

where Δ*G*_xy_, Δ*G*_x′y_, Δ*G*_xy′_, and Δ*G*_x′y′_ are the folding free energy for the wild type protein *xy*, single mutants *x*′*y* and *y*′*x*, and the double mutant *x*′*y*′, respectively. The symbols *x* and *y* denote the aliphatic and aromatic side chains in the C−H∙∙∙π or N−H∙∙∙π pair. This expression can be defined for both GB3 and Δ + PHS proteins.

### Triple mutant box analysis

Two double mutant cycles can be combined to produce a TMB, which can be used for quantification of cooperative effects. Extensive studies have been performed by Hunter and co-workers using triple mutant box experiments to evaluate cooperativity in non-covalent interactions^28,39^. Double mutants of GB3 (L5V-I7V, L5V-T16A, L5V-T18A, I7V-T16A, L5V-N37A, I7V-N37A, and T16A-N37A) and Δ + PHS (L25V-V74A, L25V-I92V and L74A-I92V) were used to set TMBs. All of these double mutant proteins could be expressed except L5V-I7V of GB3. Triple mutants were prepared, including L5V-T16A-Y33L, L5V-T18A-F30L, I7V-T16A-Y33L, L5V-N37A-Y33L, I7V-N37A-Y33L, T16A-N37A-Y33L, and T16A-N37A-F33L for GB3, and L25V-V74A-F34L, L25V-F34L-I92V, and V74A-I92V-F34L for Δ + PHS. These mutants were used to quantify the cooperativity in C−H∙∙∙π∙∙∙C−H∙∙∙π interactions and C−H∙∙∙π∙∙∙N−H∙∙∙π interactions. The folding free energy for each mutant was measured using the same method mentioned above. The cooperativity energy was then calculated using:

5 $$\begin{array}{ccc}{\Delta \Delta \Delta {\rm{G}}}_{coop} & = & {\Delta \Delta {\rm{G}}}_{xyz}-{\Delta \Delta {\rm{G}}}_{xyz^{\prime} }\\  & = & (\Delta {G}_{xyz}-\Delta {G}_{x^{\prime} yz}-\Delta {G}_{xy^{\prime} z}+\Delta {G}_{x^{\prime} y^{\prime} z})-(\Delta {G}_{xyz^{\prime} }-\Delta {G}_{x^{\prime} yz^{\prime} }-\Delta {G}_{xy^{\prime} z^{\prime} }+\Delta {G}_{x^{\prime} y^{\prime} z^{\prime} })\end{array}$$

where *y* represents the aromatic residue, *x* and *z* represent nonaromatic residues, *∆G*_xyz_, ∆*G*_x′yz_, ∆*G*_xy′z_, ∆*G*_xyz′_, ∆*G*_x′y′z_, ∆*G*_x′yz′_, ∆*G*_xy′z′_, and ∆*G*_x′y′z′_ are the folding free energy of the wild type protein *xyz*, single mutants *x*′*yz*, *xy*′*z* and *xyz*′, double mutants *x*′*y*′*z*, *x*′*yz*′, *xy*′*z*′ and triple mutants *x*′*y*′*z*′, respectively.

### Molecular dynamics simulations

MD simulations were performed using the GROMACS 4.5 package^40^ with Amber99sb^29^, Charmm27^30^, or Gromos53a6^31^ force fields. The structures of all variants of GB3 and Δ + PHS were produced by FoldX^41^ with the protein backbone fixed. Each protein was solvated by adding 10.0 Å TIP3P water^42^ (or SPC water when the Gromos53a6 force field was used) in a rectangular box, and counter ions were used to neutralize the system. 500,000 steps of energy minimization followed by 1 ns MD simulation at constant pressure (1 atm) and temperature (303 K) were performed to equilibrate the system before the production running. Three 10 ns MD production runs with different random starting velocities were performed with snapshots saved every 50 ps which were then used in the data analysis and error estimation. All backbone heavy atoms are restrained in the equilibrium and production runs. Temperature was regulated by a modified Berendsen thermostat^43^ and pressure was controlled by the extended ensemble Parrinello-Rahman approach^44,45^. The long-range electrostatic interactions were evaluated by the Particle mesh Ewald method^46,47^. The nonbonded pair list cutoff was 10 Å and the list was updated every 10 fs. The LINCS algorithm^48^ was used to constrain all bonds linked to hydrogen in the protein, whereas the SETTLE algorithm^49^ was used to constrain bonds and angles of water molecules, allowing a time step of 2 fs. In the energy decomposition analysis, only the interaction energy between the paired residues of C−H∙∙∙π or N−H∙∙∙π was calculated. The computational interaction energy ΔΔ*E* was calculated by,

6 $$\Delta {E}_{xy}={E}_{xy}=\frac{{E}_{xy-coul}}{\varepsilon }+{E}_{xy-LJ}$$

7 $$\Delta \Delta E=\Delta {E}_{xy}-\Delta {E}_{x^{\prime} y}-\Delta {E}_{xy\text{'}}+\Delta {E}_{x^{\prime} y^{\prime} }$$

where Δ*E*_xy_, Δ*E*_x′y_, Δ*E*_xy′_, and Δ*E*_x′y′_ are the x−y interaction energy in the wild type protein, x′−y in the single mutant *x*′*y*, x−y′ in the single mutant *y*′*x*, and *x*′−*y*′ in the double mutant *x*′*y*′, respectively. The symbols *x* and *y* are the same as those in Eq. 4. An effective dielectric constant *ε* of 4.0 was used for electrostatic interaction energy calculations. The computational cooperativity energy ΔΔΔ*E* was calculated by,

8 $$\Delta {E}_{xyz}={E}_{xy}+{E}_{yz}+{E}_{xz}$$

9 $$\begin{array}{rcl}\Delta \Delta \Delta E & = & \Delta \Delta {E}_{xyz}-{\Delta \Delta {\rm{E}}}_{xyz^{\prime} }\\  & = & (\Delta {E}_{xyz}-\Delta {E}_{x^{\prime} yz}-\Delta {E}_{xy^{\prime} z}+\Delta {E}_{x^{\prime} y^{\prime} z})-(\Delta {E}_{xyz^{\prime} }-\Delta {E}_{x^{\prime} yz^{\prime} }-\Delta {E}_{xy^{\prime} z^{\prime} }+\Delta {E}_{x^{\prime} y^{\prime} z^{\prime} })\end{array}$$

where *y* represents the aromatic residue, *x* and *z* represent nonaromatic residues, *∆E*_*xyz*_, ∆*E*_x′yz_, ∆*E*_xy′z_, ∆*E*_xyz′_, ∆*E*_x′y′z_, ∆*E*_x′yz′_, ∆*E*_xy′z′_, and ∆*E*_x′y′z′_ are the interaction energy of *x*−*y*−*z*, *x*′−*y*−*z*, *x*−*y*′−*z*, *x*−*y*−*z*′, *x*′−*y*′−*z*, *x*′−*y*−*z*′, *x*−*y*′−*z*′, and *x*′−*y*′−*z*′ in the wild type protein *xyz*, single mutants *x*′*yz*, *xy*′*z* and *xyz*′, double mutants *x*′*y*′*z*, *x*′*yz*′, *xy*′*z*′ and triple mutants *x*′*y*′*z*′, respectively.

### QM calculations

Two methane and one benzene molecules were built to model the cooperativity of C−H∙∙∙π∙∙∙C−H∙∙∙π. The geometries of the two models, MMB and MBM, were optimized at the MP2/6-31 + G(d,p)^50^ level. The energy calculations were performed at the MP2/aug-cc-pvtz^34^ level. All the calculations were done using the Gaussian 09 software^51^.

## Supplementary information

Supplementary information.
