# Supplementary material for: Dissecting C−H∙∙∙π and N−H∙∙∙π Interactions in Two Proteins Using a Combined Experimental and Computational Approach
Source: Sci Rep. 2019 Dec 27;9:20149. doi: 10.1038/s41598-019-56607-4 (PMC6934659; doi:10.1038/s41598-019-56607-4)
Supplement: Supplementary file 1 — Supplementary information. [file 41598_2019_56607_MOESM1_ESM.docx]

Dissecting CH−π and NH−π Interactions in Two Proteins using a Combined Experimental and Computational Approach

Jia Wang,^†,‡,§^ and Lishan Yao^*, †,‡^

^†^ Key Laboratory of Biofuels and ^‡^ Shandong Provincial Key Laboratory of Synthetic Biology, Qingdao Institute of Bioenergy and Bioprocess Technology, Chinese Academy of Sciences, Qingdao, 266101, China.

^§^ University of Chinese Academy of Sciences, Beijing, 100049, China

Table S1. Thermodynamic parameters yielded from fitting of GB3 and Δ+PHS denaturation to eq. 1.

|  | D50% | M values |
| --- | --- | --- |
| GB3 | 2.20 ± 0.01 | 1.53 ±0.04 |
| GB3_L5V | 0.85 ± 0.01 | 2.42 ± 0.10 |
| GB3_I7V | 1.58 ± 0.02 | 1.51 ± 0.05 |
| GB3_T16A | 2.07 ± 0.01 | 1.61 ± 0.03 |
| GB3_T18A | 1.91 ± 0.01 | 1.83 ± 0.03 |
| GB3_F30L | 1.55 ± 0.01 | 1.83 ± 0.02 |
| GB3_Y33L | 1.67 ± 0.01 | 2.03 ± 0.07 |
| GB3_Y33F | 2.03 ± 0.01 | 1.61 ± 0.05 |
| GB3_N37A | 2.25 ± 0.01 | 1.62 ± 0.03 |
| GB3_L5VF30L | 0.58 ± 0.02 | 3.00 ± 0.20 |
| GB3_T18AF30L | 1.30 ± 0.01 | 2.18 ± 0.09 |
| GB3_L5VY33L | 0.63 ± 0.02 | 2.88 ± 0.19 |
| GB3_I7VY33L | 1.12 ± 0.01 | 1.97 ± 0.06 |
| GB3_T16AY33L | 1.85 ± 0.01 | 1.71 ± 0.05 |
| GB3_N37AY33L | 2.04 ± 0.01 | 1.94 ± 0.06 |
| GB3_T16A_Y33F | 1.89 ± 0.01 | 1.55 ± 0.03 |
| GB3_N37A_Y33F | 2.01 ± 0.01 | 1.59 ± 0.04 |
| Δ+PHS | 2.28 ± 0.01 | 3.88 ± 0.13 |
| Δ+PHS_L25V | 1.81 ± 0.01 | 3.46 ± 0.13 |
| Δ+PHS_F34L | 1.76 ± 0.01 | 3.97 ± 0.17 |
| Δ+PHS_V74A | 1.58 ± 0.01 | 4.35 ± 0.20 |
| Δ+PHS_I92V | 2.15 ± 0.01 | 3.07 ± 0.15 |
| Δ+PHS_L25VF34L | 1.39 ± 0.01 | 3.97 ± 0.16 |
| Δ+PHS_F34LV74A | 1.19 ± 0.01 | 4.88 ± 0.11 |
| Δ+PHS_I92VF34L | 1.74 ± 0.01 | 3.96 ± 0.18 |

*^a.^* ΔΔG_D50%_ = ([*D*]_50%_(WT)− [*D*]_50%_(mut)) × <*m*>, where <m> is the average of the *m* values.

Table S2. Experimental and calculated cooperativity between XH−π interactions

|  | Experimental ∆ΔΔ*G*_coop_ (kcal/mol) | Calculated ∆ΔΔ*E* (kcal/mol) | |
| --- | --- | --- | --- |
| L5−F30−T18 | −0.16 ± 0.08 | −1.44 ± 0.05 |  |
| L5−Y33−T16 | −0.40 ± 0.08 | −1.91 ± 0.06 |  |
| I7−Y33−T16 | −0.44 ± 0.08 | −0.97 ± 0.02 |  |
| L5−Y33−N37 | −0.32 ± 0.09 | −0.29 ± 0.05 |  |
| I7−Y33−N37 | −0.24 ± 0.08 | −1.31 ± 0.04 |  |
| T16−Y33−N37 | 0.16 ± 0.08 | -0.63 ± 0.04 |  |
| T16−F33−N37 | −0.55 ± 0.08 | −1.01 ± 0.08 |  |
| L25−F34−V74 | 0.30 ± 0.07 | 1.22 ± 0.06 |  |
| L25−F34−I92 | 0.37 ± 0.10 | 1.00 ± 0.07 |  |
| V74−F34−I92 | 0.04 ± 0.09 | 0.22 ± 0.16 |  |
